# Classifying Maxillary Sinuses of Polish Patients for Sinus Lift: A Pilot Study

**DOI:** 10.3390/dj12020035

**Published:** 2024-02-05

**Authors:** Radosław Jadach, Farah Asa’ad, Giulio Rasperini, Karolina Osypko

**Affiliations:** 1 Dental Salon, Oral Surgery Academy, Horbaczewskiego 53a, 54-130 Wrocław, Poland; radek.jadach@gmail.com; 2 Department of Biomaterials, Institute of Clinical Sciences, The Sahlgrenska Academy at University of Gothenburg, 413 90 Göteborg, Sweden; farah.asaad@gu.se; 3 Department of Oral Biochemistry, Institute of Odontology, The Sahlgrenska Academy at University of Gothenburg, 413 90 Göteborg, Sweden; 4 Department of Biomedical, Surgical and Dental Sciences, University of Milan, 20122 Milan, Italy; giulio.rasperini@unimi.it; 5 Foundation IRCCS Ca’ Granda Policlinic, 20122 Milan, Italy; 6 Platinum Clinic, Księcia Witolda 49, 50-202 Wrocław, Poland

**Keywords:** sinus floor augmentation, maxillary sinus, palatal thickness, CBCT, anatomy, classification, palatal access

## Abstract

To date, there is no systematic anatomical classification available that could help clinicians in choosing between the lateral and palatal approach in sinus lift procedures. The aim was to provide a simple-to-use and memorable classification of the maxillary sinus concerning the thickness of lateral and palatal walls to facilitate the most adequate choice for the window location during direct sinus floor elevation. Cone beam computed tomography scans were consecutively obtained for 200 maxillary sinuses of patients needing dental implant placement with potential maxillary sinus augmentation. The thickness and height of the alveolar bone of the lateral and palatal walls of the maxillary sinuses were assessed. Four variants were distinguished. Class 0: an adequate sub-sinus residual bone height; without the need for sinus floor augmentation. Classes 1–3 had a reduced sub-sinus residual bone height. Class 1: a thinner lateral than palatal sinus wall. Class 2 (the most frequent; 49%): the comparable thickness of both walls in which either lateral, palatal, or crestal window osteotomies can be applied. Class 3 (the least frequent; 3%): a thinner palatal sinus wall in comparison to the lateral wall. The presented anatomical classification simplifies the decision-making process of choosing the most adequate window location and osteotomy technique.

## 1. Introduction

In the posterior maxilla, continuous bone remodeling and a lack of functional loading lead to pneumatization of the maxillary sinus, in which there is a massive reduction in the residual vertical bone height [1,2]. In this scenario, several treatment options have been proposed to conquer the inadequate amount of bone in the posterior maxillae, ranging from conservative to invasive. Among the conservative procedures are the placement of short dental implants, tilted implants mesial or distal to the sinus cavity (if these areas bear a sufficient amount of bone), and the concept of a shortened dental arch [3].

However, in certain clinical situations, maxillary sinus elevation (sinus lifting) and the placement of bone-graft material become obligatory whenever dental implant placement is intended by utilizing different techniques based on the sub-sinus alveolar bone height. When the remaining bone height is 5–6 mm and the placement of short dental implants is not intended, sinus lifting with an indirect approach through the alveolar crest is applied; bone is compacted laterally and apically around the implant site by using osteotomes of progressively increasing diameter [4]. Meanwhile, if the residual bone height is less than the previously designated amount [5], the window osteotomy technique becomes the method of choice, in which direct visualization and manipulation of the Schneiderian membrane are possible. The window osteotomy technique usually employs the lateral approach, which provides buccal access to the lateral sinus wall [6].

Although the lateral approach is widely accepted in implant surgery and oral rehabilitation [7], it is associated with various complications, such as perforation of the Schneiderian membrane [8] transient maxillary sinusitis [9], post-operative swelling, and hematoma formation in the cheek and under the eye [6], all of which could compromise the overall satisfaction of the patient. Accordingly, direct lifting of the maxillary sinus floor from either the palatal side [10] or the alveolar crest [11] has been introduced in an attempt to overcome the side effects encountered with the conventional approach.

In a comparative clinical study by Stübinger, although the incidence of perforation of the Schneiderian membrane was equal for the lateral and palatal approaches (19%), the latter was favorable in terms of soft tissue management and post-operative sequelae because of the eliminated need for releasing incisions beyond the mucogingival line on the buccal side [12] and the detachment of muscles from their insertion, consequently keeping the vestibular anatomy unaltered. Also, the intimate interconnection of the palatal mucosa with the underlying periosteum allows for an exact re-adaptation of the flap on the palatal bone wall, which prevents the distortion of soft tissue structures [13]. Such stability of the palatal mucosa reduces the risk of post-operative swelling and hematoma. In addition, thick palatal mucosa might make this approach advantageous in people who smoke and those with diabetes, where wound healing is compromised [14]. In a study by Rahpeyma and Khajehahmadi [15], palatal access is recommended in patients with a deep palatonasal recess, heavy buccal vestibule scarring, a thick buccal bone, and also for reentry augmentation [16,17].

Nevertheless, the palatal approach encompasses several disadvantages, mainly for the clinician, in terms of difficult access, limited vision, a lack of specially designed curved instruments, and the presence of the greater palatine artery, which can be injured if an initial vertical releasing incision is intended, resulting in intra-operative bleeding and an increased risk for palato-maxillary sinus fistula, which is difficult to manage.

Concerning direct lifting of the maxillary sinus floor from the alveolar crest, it might provide the possibility for the clinician to perform a minimally invasive surgery with direct visual control and is reportedly associated with a very low incidence of Schneiderian membrane perforation (6.67%) [11]. Due to the accompanying minimal surgical trauma, this approach might favor revascularization and minimize post-operative complications, although further investigations are needed to validate current conclusions.

By now, the documented cases of alternative approaches (such as palatal access) do not provide any universal qualification criteria, which could be considered practical guidelines for surgeons [12,15].

From this perspective and the potential advantages of palatal access to the maxillary sinus, this study was designed to create a simple classification as a tool for an improved and simplified decision-making process before planning surgery and to unleash the discussion about the possibilities of palatal access in general.

The analysis of CBCT scans is an acknowledged tool to assess the condition of the maxillary sinus and bone thickness [18,19]; thus, it has been chosen by authors as an appropriate tool to conduct this study. Moreover, none of the patients underwent CBCT examination for the sole purpose of this study, as the existing database of CBCT examinations (conducted for different dental purposes) was used.

This is a pilot study, as further research on palatal access for the sinus lift procedure is being conducted and will be extensively summarized in another study.

## 2. Materials and Methods

The analyzed data were based on a cone beam computed tomography (CBCT) database of patients attending a private dental practice in Poland. The database was randomly searched until a sample of 200 maxillary sinuses was collected. The inclusion criteria were as follows:

- An edentulous area in the posterior maxilla with a potential sinus floor augmentation;
- No radiological signs of maxillary sinus disease (i.e., a widened Schneiderian membrane, radiological image typical of chronic sinusitis, foreign bodies);
- No history of a direct osteotomy (e.g., osteotomy to remove a foreign body from the maxillary sinus);
- The last tooth extraction or other surgical intervention in the posterior maxilla was performed no earlier than 12 months ago at the time of data collection.

Three types of measurements on the coronal view scans were taken:

1. Sub-sinus residual bone height: measured as the distance from the top of the alveolar ridge to the floor of the sinus to establish whether sinus floor augmentation is necessary before implant placement.
2. Thickness of the lateral wall: measured on the level of assumed window osteotomy, at the thinnest point.
3. Thickness of the palatal wall: measured in the middle of the wall, also at the thinnest point.

**Figure 1:**
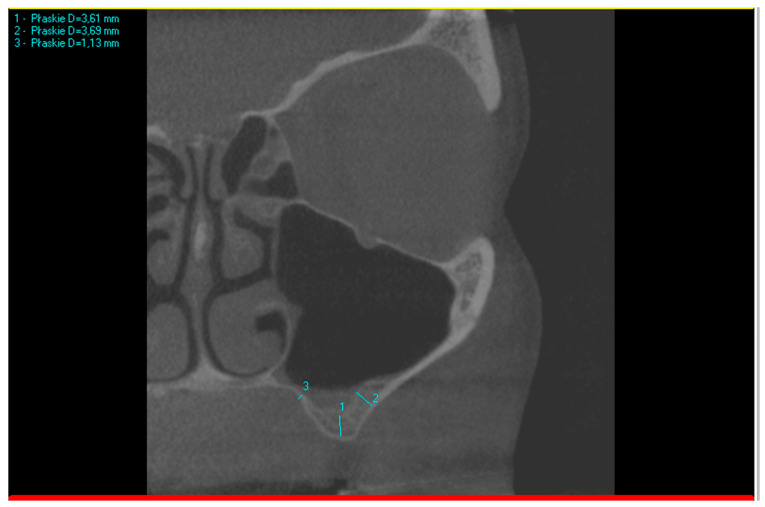
Example of three measurements taken on a CBCT scan. Measurement no. 1 is sub-sinus residual bone height, measurement no. 2 is the thickness of the lateral wall, and measurement no. 3 is the thickness of the palatal wall.

The three measurement points on the CBCT scans are illustrated in Figure 1.

When the sub-sinus residual bone height was at least 5 mm or more [20], cases were indicated as sufficient for immediate implant placement or indirect sinus lift.

When comparing the thickness of the lateral and palatal walls in patients who needed direct sinus floor augmentation, if the difference between the two wall measurements was less than 100% of the smaller measurement, their thickness was considered comparable. In the example shown in Figure 1, measurement no. 1 (3.61 mm) presents insufficient bone height for implant placement. Based on that, the following subsequent measurements were taken: no. 2 (3.69 mm) and 3 (1.13 mm). The difference between the thicker wall (3.69 mm) and the thinner wall (1.13 mm) was 2.56 mm, which is more than 1.13 mm (100% of the thinner wall). In this case, the walls cannot be described as of a similar thickness.

Data were collected simultaneously by two researchers (K.O. and R.J.) and entered into an Excel spreadsheet.

Measurements were conducted in an imaging system dedicated to CBCT radiographs (Field of View: 14 cm diameter × 8.5 cm height, 0.2 mm voxel, GXCB-500 HD, Gendex, Hatfield, PA, USA), as CBCT can be used successfully to determine the thickness of alveolar bone [18] and sinus presurgical evaluation [19].

All participants gave their written consent after they were verbally informed about the study, explaining its protocol and objectives.

This study was conducted according to the STROBE guidelines/checklist.

## 3. Results

This study included 200 maxillary sinuses from patients with an age range between 33 and 69 years old (mean age = 51 years).

**Figure 2:**
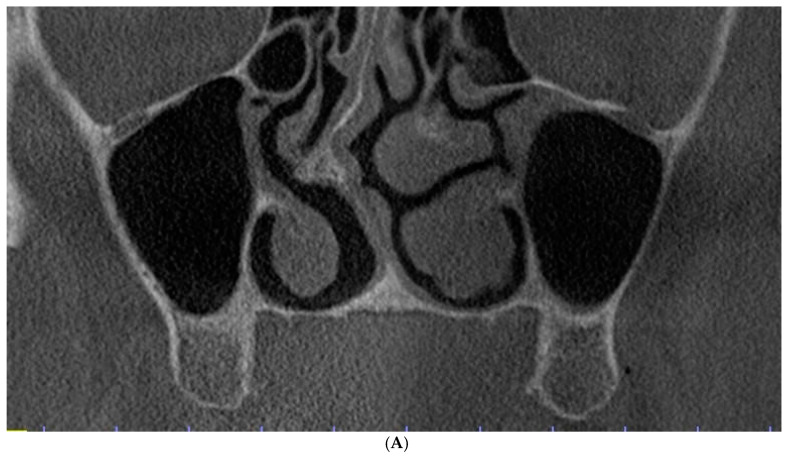

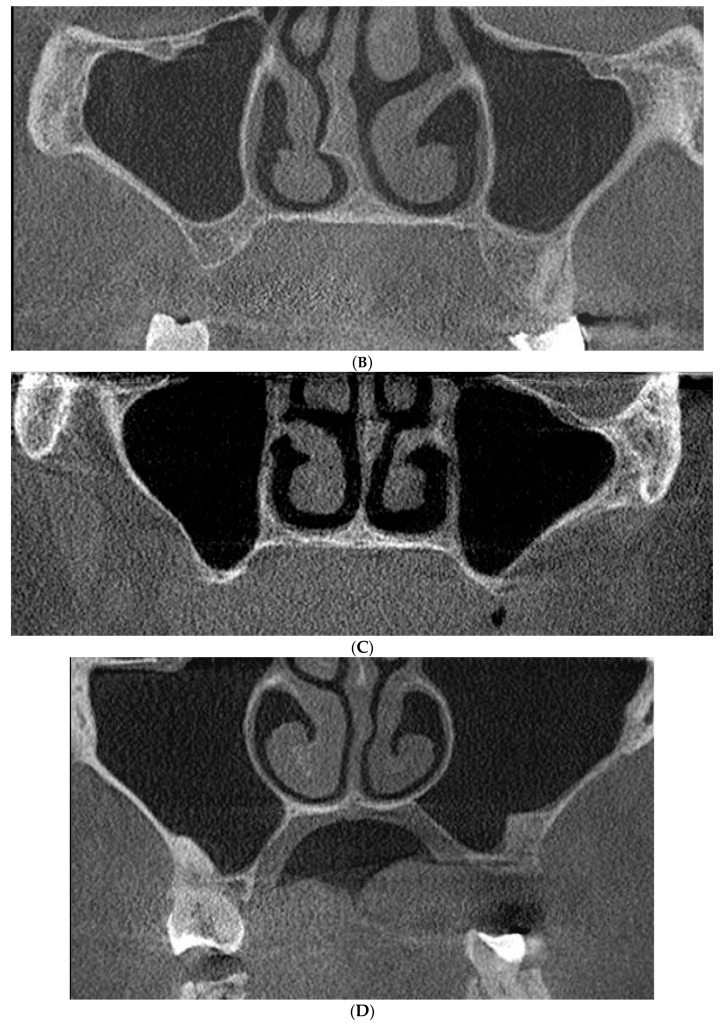
Evaluation of the maxillary sinus based on cone beam computed tomography (CBCT) scans in the coronal view. (**A**) Class 0: Adequate sub-sinus alveolar bone height. (**B**) Class 1: Reduced sub-sinus alveolar bone height with a thin lateral wall. (**C**) Class 2: Reduced sub-sinus alveolar bone height with comparable thickness of the lateral and palatal walls. (**D**) Class 3: Reduced sub-sinus alveolar bone height with a thin palatal wall.

Data analysis of the coronal view displayed four variants in the thickness of the lateral and palatal walls of the maxillary sinus with the corresponding appropriate window osteotomy technique, as follow:

Class 0: There is an adequate sub-sinus residual bone height, which is not indicated for direct maxillary sinus elevation (Figure 2A). The incidence of this finding was 4%.

Class 1: There is a reduced sub-sinus residual bone height with a thinner lateral sinus wall in comparison to the palatal wall (Figure 2B), which can be indicated for the lateral window osteotomy technique because buccal access is presumably easier. The crestal window osteotomy technique can also be applied. The incidence of such variant was common (44%).

Class 2: There is a reduced sub-sinus residual bone height with the lateral and palatal sinus walls having a comparable thickness (Figure 2C), which can be indicated for either lateral or palatal window osteotomy. The crestal window osteotomy technique can be utilized as well. This variant was evident in almost half of the obtained scans (49%).

Class 3: There is a reduced sub-sinus residual bone height with a thinner palatal sinus wall in comparison to the lateral wall (Figure 1 and Figure 2D), which can be indicated for the palatal window osteotomy technique or crestal window osteotomy technique. This was the most uncommon variant (3%), which might be logical as the palatal bone is usually denser and thicker [21].

A summary of the anatomical evaluation of the maxillary sinus is presented in Table 1.

## 4. Discussion

There are various surgical approaches reported in the literature: indirect sinus lifting by crestal approach and direct sinus lifting, either by “lateral” window osteotomy, “palatal” window osteotomy, or “crestal window osteotomy”.

The choice between direct and indirect sinus lifting is mainly dictated by the sub-sinus residual bone height. On the other hand, there are no established guidelines or criteria for the selection between the lateral, crestal, or palatal window osteotomy techniques, which could be attributed to the progressive emergence of interest in the latter one.

Although palatal and crestal window direct osteotomies reportedly have favorable outcomes in terms of soft tissue management [11,12,13,17], both techniques still have a limited number of available clinical studies. Accordingly, we have provided a radiographic analysis that might help in investigating various window location options. Furthermore, the case series of the entire patient base (200 sinuses), randomized based on anatomy; the results; and complications could be used to assess future “guidelines” in the selection between different direct sinus lifting techniques.

The analysis of the obtained CBCT scans revealed four variants in the thickness of the lateral and palatal walls of the maxillary sinus, presented in Table 1. It was also shown that the same patient might experience different anatomical variants between the right and left sides.

The most common variant was the comparable thickness of the lateral and palatal sinus walls (Figure 2C), in which either the lateral, palatal, or crestal window osteotomies can be applied (49%).

Since almost half of the cases might be eligible for different approaches in direct sinus lifting, it is meaningful to further inspect the alternative window osteotomy techniques, also in comparison to the traditional, lateral approach. Various prospective studies are still needed to validate and confirm these methods before being applied as “routine” procedures in direct sinus lifting. The current radiographic evaluation of the maxillary sinus might be useful for such purposes.

In a similar study [22], maxillary sinus anatomy is divided into three classes, depending on the depth of the alveolar process: class I (sinus floor/alveolar process above the hard palate), class II (0–6 mm below the hard palate), and class III (>6 mm below the hard palate) [22]. In that study, class I is recommended only for the lateral approach (on the premise that an alveolar ridge is insufficient for implant placement and bone augmentation is needed). Furthermore, class II is recommended for the lateral approach, and class III is suitable for all three approaches (lateral, palatal, and crestal). That study highlights sinus depth as another aspect worth taking cognizance of, as a deep sinus provides more area for making a palatal window.

According to Wagner et al., the palatal approach is technically possible in 93,6% of cases [23], although this sole possibility does not indicate the best or most reliable measure, and clinicians need the tools to simplify the decision-making process when choosing the most adequate location for the osteotomy window. As such, the new classification proposed in this study can be helpful to clinicians in their decision-making process between lateral and palatal approaches.

To minimize the risk of potential measurement errors, the overall number of analyzed sinuses could be greater. Moreover, more researchers could be included and independently collect data to obtain more averaged results.

## 5. Conclusions

Based on the radiographic analysis of the maxillary sinus with cone beam computed tomography (CBCT) scans, there are four variants of the bone thickness of the lateral and palatal walls of the maxillary sinus (Table 1). These findings permitted the creation of an uncomplicated and memorable classification which can simplify the decision-making algorithm of where to plan the most adequate osteotomy window location—on the lateral or palatal wall. This study may encourage future research of different direct sinus lifting surgical techniques with scientifically proven guidelines and contra/indications.

## Figures and Tables

**Table 1:** Incidence rate and the indicated surgical procedure in the four categories of anatomical evaluation of the maxillary sinus.

Class	0	1	2	3
Incidence Rate	4%	44%	49%	3%
Sinus Lifting Procedure	Not indicated for direct sinus lift surgery	Can be indicated for crestal or lateral window sinus lift surgery	Can be indicated for crestal, lateral, or palatal window sinus lift surgery	Can be indicated for crestal or palatal window sinus lift surgery

## Data Availability

The data that support the findings of this study are available from the corresponding author upon request.
